# Influences of Dog Attachment and Dog Walking on Reducing Loneliness during the COVID-19 Pandemic in Korea

**DOI:** 10.3390/ani12040483

**Published:** 2022-02-16

**Authors:** Hyung-Sook Lee, Jin-Gyeoung Song, Jeong-Yeon Lee

**Affiliations:** Department of Landscape Architecture, Kyungpook National University, Daegu 41566, Korea; luvpopconi@knu.ac.kr (J.-G.S.); hyo04031@knu.ac.kr (J.-Y.L.)

**Keywords:** companion animals, dog walking, dog attachment, loneliness, COVID-19

## Abstract

**Simple Summary:**

Even before the emergence of COVID-19, animal companionship was gaining popularity in Korea as a strategy to reduce loneliness and isolation caused by a rapidly aging population and an increase in one-person households. Many dog owners have experienced the emotional and physical benefits that dog ownership provides during the COVID-19 period. This study examined the relationship between dog attachment, dog walking and loneliness during times of widespread isolation. An online, cross-sectional survey for dog owners was conducted in the fall of 2020. Voluntary participants responded to questionnaires about their relationship with dogs, dog attachment, and dog walking during COVID-19. Our study suggests that dog walking has no direct effect on reducing loneliness but the relationship between dog walking and loneliness might be mediated by attachment. Due to the strict social distancing guidelines during the pandemic, dog walking would not allow opportunities for conversation with other people, but it seems that spending time outside with dogs strengthens the bonds between humans and companion dogs, alleviating loneliness.

**Abstract:**

The COVID-19 pandemic has changed people’s lives and increased their vulnerability to physical and mental health hazards. While Korea has avoided nationwide lockdown measures since the COVID-19 outbreak, the prolonged restrictions and social isolation measures have resulted in detrimental psychological effects, such as increased anxiety, boredom, and loneliness. The present study investigated dog attachment and changes in dog walking during the COVID-19 pandemic and the effects of dog attachment and dog walking on the loneliness of Korean dog owners. An online, cross-sectional survey was conducted in the fall of 2021 in which 249 dog owners responded to questionnaires that asked questions about dog attachment, their perception of dog walking, and their feelings of loneliness during the COVID-19 pandemic. Most dog owners responded that they spent more time with their dogs and developed a stronger bond with them during the pandemic. Additionally, respondents stated that they walked their dogs more often than they did before COVID-19 and that their dogs aided in reducing loneliness. We found that dog walking directly affected attachment and indirectly influenced the loneliness of dog owners. Further research is required to determine how dog walking impacts positive psychological effects and promote dog walking.

## 1. Introduction

The COVID-19 pandemic has changed people’s lives and increased their exposure to physical and mental health risk factors. While Korea tried to avoid nationwide strict lockdown measures in the aftermath of the COVID-19 outbreak, the prolonged restrictions and social isolation have caused adverse psychological effects, such as anxiety, boredom, and loneliness [1]. According to the 2021 Ministry of Health and Welfare’s COVID-19 National Mental Health Survey, the amount of people at risk for developing depression has increased sixfold to 22.8%, up from 3.8% before the outbreak [2]. COVID-19 was found to be more detrimental to young people. Specifically, 30.0% of individuals in their twenties and 30.5% in their thirties, and 14.4% of those in their sixties are considered to be in groups at risk of developing depression [2]. As social isolation has become prevalent during the pandemic, dog adoptions and the demand for adoptable dogs have increased globally [3]; Korea was not the exception. According to the annual survey of the Ministry of Agriculture, Food and Rural Affairs in 2020, 27.7% of Korean households (6.38 million) own at least one companion animal, up 7.4% from the previous years’ 5.91 million households [4]. Even before COVID-19, animal companionship was gaining popularity in Korea as a strategy to reduce loneliness and isolation caused by a rapidly aging population and one-person households. Many dog owners have experienced the emotional and physical assistance that dog ownership provides during times of widespread isolation and loneliness.

### 1.1. Benefits of Dog Walking

Dog walking around neighborhoods and visiting open spaces with dogs were feasible and popular activities among Korean dog owners since they were permitted even during the height of the pandemic. There is a body of research on the physical and mental health benefits. Dog walking can serve as a motivation for both dog owners and dogs to regularly engage in walking exercises and a means of ensuring that urban residents continue to maintain their health [5]. Dog owners engage in more physical activities and do so more frequently than people who are not dog owners [6]. Regular physical activity through exercise and walking with dogs is associated with preventing non-inflammatory diseases such as heart disease and diabetes and maintaining health [7]. Bauman et al. predicted that walking a dog for at least 150 min a week can prevent 9% of coronary heart disease and that if all owners walk their dogs, they can prevent diseases and reduce medical costs by around 175 million Australian dollar per year [8]. In addition, the obesity rate of adult caregivers walking dogs was 17%, while the rate of obesity in those who did not participate in dog walking was 28% [9]. These results indicate that dog walking helps maintain proper weight and prevent diseases. The advantages of health promotion due to dog walking can be more effective, especially for the elderly and vulnerable groups with poor health. Older adult dog owners participated in outdoor walks more than those who did not raise dogs [10] and were more likely to meet the recommended daily physical activity recommendations [11]. According to a study by Gretebeck, elderly dog owners who walk their dogs at least three times a week were twice as likely to walk for more than 150 min every week as compared with non-dog owners [12]. Walking a dog helps individuals feel fewer negative emotions and promotes emotional stability through social and emotional support [13]. In particular, outdoor activities and walks with dogs help maintain mental health by promoting exchanges with others and reducing depression [14].

### 1.2. Dog Walking and Loneliness

In terms of the association between dog walking and loneliness, the role of dogs in catalyzing owners to interact with other people is associated with reduced loneliness [15]. Carr et al. found that older adults with high social consequences experienced significant increases in loneliness, but daily dog walking reduced feelings of loneliness [16]. Researchers have explored the possible beneficial effects of dog walking concerning loneliness. The social interactions that occur while dog walking help reduce feelings of loneliness and stress levels [17,18,19]; dog walking and the relationships that dog walkers develop with other dog walkers may help reduce their stress levels. Antonacopoulos and Pychyl showed that if dog walkers develop a strong sense of community through dog walking, it could reduce their feelings of loneliness [17]. During COVID-19, however, people are much less likely to interact with other people encountered while dog walking due to physical distancing orders [20]. Therefore, the association between dog walking and reduced loneliness through social contact might be weak during the COVID-19 pandemic.

### 1.3. Dog Walking and Attachment

Numerous studies have examined the relationship between dog walking and attachment. Andreassen et al. investigated the relationship between dog owners’ attachment to their dogs and the amount of time they spent walking them [21]. Increased attachment to dogs was positively associated with physical exercise, such as walking, which led to better health [22]. Furthermore, those who spent more time walking with their dogs reported they could have greater attachments and intimacy simply due to their time together [23]. Scandurra et al.’s empirical study showed that the human/dog attachment bond was not improved by training, suggesting that it independently develops by spending their daily lives together [24].

Regarding the relationship between attachment and loneliness, it was found that the stronger the attachment to the companion animal, the lower the chance of depression [25,26]. However, few studies have examined the relationship between dog walking and reduced loneliness. Since social interactions during dog walking are less likely to occur during the pandemic, we assumed that dog attachment might impact the relationship between dog walking and reduced loneliness. Therefore, the objectives of this study were (1) to discern any changes in dog attachment and dog walking during the pandemic period in Korea, and (2) to investigate the relationship between dog attachment and dog walking and their effect on loneliness in dog owners.

## 2. Materials and Methods

### 2.1. Data Collection

An online, cross-sectional survey for dog owners was conducted in the fall of 2021. Before the formal survey, a preliminary trial of the questionnaire involving 11 dog owners was conducted to eliminate or adjust any ambiguous or misleading items. The pre-test allowed the online survey format to be finetuned and the questions reworded. This study was reviewed and approved by the University’s Institutional Review Board (IRB KNU-2021-0156). In order to recruit survey participants, notices about the survey with QR codes were displayed on bulletin boards in veterinary clinics, dog cafes, and dog shops in Daegu, Korea.

Additionally, kennel clubs were provided with a survey link and QR code for the online survey to be distributed to dog owners willing to participate in the study. Potential participants were informed that participation in the study was entirely voluntary and that scanning a QR code would direct them to an online questionnaire created using the Google Form tool. In addition, participants were informed about the study objectives and confidentiality issues.

### 2.2. Questionnaire Design

A comprehensive literature review was conducted to compile a reliable set of items to measure the constructs and test the proposed model. Multiple items from the Dogs and WalkinG Survey (DAWGS) [27] and Dogs and Physical Activity (DAPA) [28] were used to investigate dog ownership status, dog walking behavior, and perceived outcomes of dog walking. The DAWGS is a psychometrically sound instrument related to interpersonal correlations of dog walking, and items confirm moderate test–retest reliability. Items from the DAPA tool were used to measure the physical activities of dog owners and essential characteristics that affect walking with dogs. The items of DAPA have high test–retest reliability and can measure dog-supportive park features, barriers to dog walking, and perceived behavioral outcomes of dog walking.

Attachment and relationship were measured with five items from the sub-scale of DAPA: “I think of my dog(s) as a family”, “I feel happier thanks to my dog(s)”, “I talk to my dog(s)”, “My dog(s) seems to know my feelings well”, and “I often play with my dog(s)”. In addition, two questions were included about any changes in their attachment during the COVID-19 pandemic: “I spend more time with my dog(s)” and “I became more attached to my dog(s)” during the pandemic. The perceived outcomes of dog walking comprise eight items (Cronbach’s α = 0.73), which were derived from the items of DAWGS, including “dog walking helps maintain my health” and “dog walking increases physical activity”. Additionally, we asked about any increases in dog walking during the pandemic and participants’ perception of the walking environment. Participants were asked to rate each item on a 5-point scale ranging from 1 (strongly disagree) to 5 (strongly agree). Simple demographic questions such as gender, age, family type, and education were collected. The questionnaire is available in the Supplementary Materials.

### 2.3. Statistics

**Figure 1:**
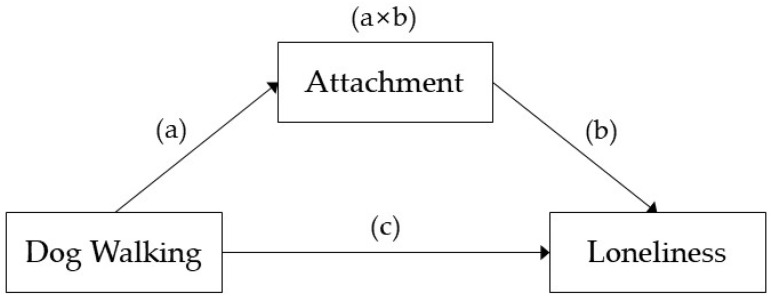
The hypothesized mediation model illustrating the predictive paths between dog walking, attachment and loneliness. Dog walking predicts attachment (Path a); attachment predicts loneliness (Path b); dog walking predicts loneliness (Path c); and attachment mediate the relationship between dog walking and loneliness (Path a × b).

The data derived from the questionnaire were systematically coded and analyzed using IBM SPSS v.25 (IBM Corp., Armonk, NY, USA). Chi-square analyses were used to determine the difference in attachment level according to participants’ gender and the frequency of dog walking. The frequency dataset was dichotomized into two groups—“once a day or more” and “less than once a day”—to verify the statistical significance of the differences between the two groups. Multiple linear regression analyses were used to determine the association between dog walking, attachment, and loneliness. Before the regression analysis, a Pearson correlation analysis was performed to examine the correlation between variables. We expected that the relationship between dog walking and loneliness might be mediated by attachment (Figure 1). We did so following the prescriptions of Zhao, Lynch, and Chen (2010), who provided an updated procedure versus the traditionally employed Baron and Kenny (1986) mediation testing procedure [29,30]. First, we expected that dog walks would significantly impact loneliness (Path c). Second, we needed to verify whether dog walking is associated with attachment (Path a). Third, attachment should affect loneliness (Path b). Fourth, when loneliness is predicted by combining dog walking and attachment (Path a × b), and if dog walking does not affect loneliness, it can be called complete mediation. Paths a, b, c, and a × b were calculated using linear regression. The significance of the mediator (attachment) was verified using the Sobel test. Finally, we used model 4 of Hayes’ (2013) PROCESS macro for SPSS version 4.0 to examine the mediating role of dog attachment between dog walking and loneliness [31]. In the model we ran, indirect effects were subjected to bootstrap analyses with 5000 bootstrap samples and a 95% confidence interval. A significant indirect effect is considered to have emerged if a bias-corrected bootstrap 95% confidence interval for the product of the paths excludes zero [32].

## 3. Results

### 3.1. Participants’ Demographics and Dog Ownership

Participants included 207 women (83.1%) and 42 men (16.9%). One-hundred-and-eight (43.4%) participants were under the age of 29, and 94 (37.8%) participants were in their thirties, accounting for nearly 80% of all respondents in their twenties and thirties (Table 1). The proportion of those who lived with their parents was 37.8%, followed by those who lived alone (26.5%) and married couples (19.3%). A total of 64.7% had completed college and 5.2% had completed graduate school. A total of 163 (65.5%) respondents own one dog, 24.5% (53) own two dogs, and 10.0% (25) own three or more dogs, with an average of 1.45 dogs. In terms of dog walking patterns, 119 (47.8%) owners take their dogs on walks more than once a day, up to four times a day. Approximately 60% of dog owners walk their dogs for 30 min to an hour, and nearly 47.0% responded that they walked their dogs around neighborhoods close to their homes during the COVID-19 pandemic.

### 3.2. Perception on Dog Relationships and Changes during the Pandemic Period

Items addressing attachment to the dog were used to investigate the perception of the relationship with the dog(s), and almost all participants stated that they believed their dog(s) was a member of the family (M = 4.9), that they felt happy (M = 4.9) and less lonely (M = 4.7) thanks to dogs (Table 2). Furthermore, most dog owners talked to their dogs (M = 4.7) or felt that their dogs sensed their feelings (M = 4.1). In addition, participants reported spending more time with their dogs during the pandemic period (M = 4.2) and becoming more attached to their dogs (M = 3.9).

### 3.3. Perceived Outcomes of Dog Walking and Changes during the Pandemic Period

Regarding dog walking, dog owners believe that it benefits not only the dog’s health (M = 4.4) but also their own health (M = 4.1) and that it encourages more physical activity (M = 4.3, Table 3). Due to social distancing during the pandemic, the benefits of opportunities to talk to other people scored relatively low (M = 3.7). Dog owners responded that they walked their dogs more often than they did before the COVID-19 pandemic (M = 3.3) and emphasized the importance of green spaces for taking a walk (M = 4.1) during the pandemic.

### 3.4. Differences of Perception by Frequency of Dog Walking

It was found that there was a relationship between dog attachment and walking frequency (Table 4). The participants who walked their dogs once a day or more responded higher on the question of frequently playing with dogs when compared with those who walked with their dogs less than once a day (*p* = 0.002). In addition, the walked once a day or more group responded that their attachment to their dog increased during the pandemic period as they spent more time with their dog during the pandemic period (*p* = 0.037). The higher the frequency of walking with a dog, the more helpful for health for both dogs and dog owners (*p* = 0.01). In addition, respondents who walked their dogs once a day or more reported that dog walks increased their physical activity significantly higher (*p* = 0.004) than those who walked them less than once a day. The more frequently they walked their dogs, the more likely they felt guilty if they did not walk them often enough (*p* = 0.013). In addition, dog walks were more frequent during the pandemic (*p* = 0.006).

### 3.5. Association of Dog Walking and Loneliness

Pearson correlations were calculated before running linear regression on dog walking and its relationship with loneliness (Table 5). As a result, dog walking showed a statistically significant positive correlation with attachment (r = 0.481, *p* < 0.01), while loneliness showed a significant positive correlation with attachment (r = 0.226, *p* < 0.01). There was no significant correlation between dog walking and loneliness. Therefore, this study attempted to verify the mediating effect of attachment with a significant correlation between dog walking and loneliness.

As shown in Table 6, the association between dog walking and attachment was significant (Path a: B = 0.454, *p* = 0.000). Loneliness was found to decrease significantly as attachment to dog increased (Path b: B = 0.124, *p* = 0.000). Dog walking is not significantly related to loneliness (Path c: B = 0.054, *p* = 0.102). A positive relationship was observed when attachment between dog walking and loneliness was mediated (Path a × b: B = 0.125, *p* = 0.002). The indirect effect of dog walking on loneliness (a × b) was statistically significant. For the indirect effect, bootstrapped 95% confidence interval (CI) did not cross zero (0.023, 0.096), which indicated that attachment partially mediated the relationship between dog walking and loneliness (B = 0.057, SE = 0.018).

## 4. Discussion

The present study investigated changes in dog attachment and dog walking patterns of Korean dog owners and the relationship between dog walking, attachment, and loneliness during the COVID-19 pandemic. The following are the research’s major findings and implications.

First, we found that companion dogs helped reduce negative feelings during the COVID-19 pandemic in Korea. Additionally, they helped maintain physical health by increasing dog walking around neighborhoods. Many studies conducted in Europe and North America during the COVID-19 lockdown phase indicate that companion animals positively impacted dog owners’ mental and physical health [20,33]. Although Korea was not under a strict nationwide lockdown during the COVID-19 pandemic, people in Korea experienced stress and hardships due to the prolonged restrictions and social isolation. As expected, many people stated they developed a stronger bond with their dogs during the COVID-19 pandemic, owing to dog owners’ increased time at home.

Second, dog walking was critical in maintaining physical and emotional mental health for both companion dogs and their owners during the COVID-19 pandemic in Korea. Over half of the respondents take dog walks more than once a day, and almost 70% take dog walks more than four times a week. Significantly, those who walked their dogs frequently experienced greater physical and psychological benefits from dog companionship than those who walked their dogs less frequently. These results could be because dog walking increases overall physical exercise and maintains regular schedules, which helps avoid negative feelings such as boredom and anxiety [34]. In addition, due to their proximity to home, the popular places for dog walking in this study were their neighborhoods and community parks, consistent with prior studies on factors promoting dog walking [35].

Third, dog walking was associated with loneliness, and this relationship was mediated by dog attachment. The potential role of catalyzing social interaction was expected to mediate the relationship between dog walking and reduced loneliness [17]. However, due to the strict social distancing during the pandemic, dog walking would not elicit possibilities for conversation with other people. Therefore, we assumed that spending time outside with dogs strengthens the bonds between humans and companion dogs, alleviating loneliness.

Dog walking can be an effective way for urban residents and their companion dogs to increase their physical activity and maintain their health. However, many dog owners in Korea cite hindrances in the way of dog walking due to insufficient places and amenities for dog walking and exercise. Providing off-leash dog parks and improving dog walking environments would encourage and promote physical activity and maximize the psychological benefits of dog walking.

There are limitations of this study. First, the results cannot be generalized because the research was undertaken during a unique period. Second, most participants were in their twenties and thirties and were relatively familiar with mobile surveys. Third, self-report response bias can occur in measuring the frequency and duration of dog walking. Given the paucity of research on dog attachment and loneliness during the COVID-19 pandemic in Asia, this study would contribute to a better understanding of the critical role of dog walking and attachment in alleviating adverse effects during the pandemic.

## 5. Conclusions

The objectives of this study were to discern any changes in dog attachment and dog walking during the COVID-19 pandemic period in Korea and investigate the relationship between dog attachment and dog walking and their impact on the loneliness of dog owners. In keeping with previous findings, dog companionship resulted in psychological benefits such as decreased loneliness and feelings of happiness for dog owners. Additionally, dog walking aided in maintaining health during the COVID-19 pandemic through regular physical activity. In particular, we found that dog walking indirectly impacted reduced loneliness by mitigating attachment. Consequently, providing open spaces and a walking environment for dog owners and their companion dogs to exercise and interact would help promote physical activity and maintain mental health during COVID-19.

## Figures and Tables

**Table 1:** Sociodemographic of online survey participants (*n* = 249).

Category		*n* (%)
Gender	Female	207 (83.1%)
	Male	42 (16.9%)
Age	29 years old or less	108 (43.4%)
	30 to 39 years	94 (37.8%)
	40 years old or more	47 (18.8%)
Family type	Single residence	66 (26.5%)
	Married couple	48 (19.3%)
	Living with parents	94 (37.8%)
	Living with children	30 (12.0%)
	Others	11 (4.4%)
Education	High school graduate or less	75 (30.1%)
	College graduate	161 (64.7%)
	Graduate school or higher	13 (5.2%)
Dogs living in the home	One dog	163 (65.5%)
	Two dogs	61 (24.5%)
	Three or more dogs	25 (10.0%)
Walking frequency	Once a day or more	119 (47.8%)
	4–6 times a week	54 (21.7%)
	2–3 times a week	49 (19.7%)
	Once a week	17 (6.8%)
	One to three times a month	10 (4.0%)
Walking time per walk	30 min or less	47 (18.9%)
	More than 30 min to 1 h	148 (59.4%)
	More than 1 h to 2 h	50 (20.1%)
	More than 2 h	4 (1.6%)

**Table 2:** The perception of the relationship with the dog.

		M	SD
Dog relationship	I think of my dog(s) as a family.	4.9	0.3
	I feel happier thanks to my dog(s).	4.9	0.4
	I feel less lonely thanks to my dog(s).	4.7	0.6
	I talk to my dog(s).	4.7	0.5
	My dog(s) seems to know my feelings well.	4.1	0.9
	I often play with my dog(s).	4.0	0.8
	During the pandemic, I spend more time with my dog(s).	4.2	1.0
	During the pandemic, I became more attached to my dog(s).	3.9	1.1

**Table 3:** The perceived outcomes of dog walking.

		M	SD
Perceived outcomes of dog walking	I also get to do more physical activities.	4.3	0.8
	It helps me maintain my health.	4.1	0.9
	It is helpful for the health of dog(s).	4.4	0.7
	It’s a break for me.	4.1	0.9
	Opportunities to talk to other people arise.	3.7	1.1
	If I don’t take a dog(s) walk often I feel guilty.	4.3	0.8
	During the pandemic, I walked my dog(s) more often.	3.3	1.1
	During the pandemic, I felt that there was not enough space to take a walk with my dog(s).	3.2	1.1
	During the pandemic, I felt the importance of the park/green area to take a walk.	4.1	1.0

**Table 4:** Differences in dog relationship and dog walk awareness between the groups.

Perception	Group	N	Mean	SD	df	*t*-Value	*p*-Value	Cohen’s d
I often play with my dog(s).	Once a day or more	119	4.1	0.8	247	3.08	0.002 ***	0.39
	Less than once a day	130	3.8	0.8				
During the pandemic period, I spend more time with my dog(s).	Once a day or more	119	4.3	0.9	247	2.09	0.037 *	0.27
	Less than once a day	130	4.1	1.0				
I also get to do more physical activities.	Once a day or more	119	4.4	0.8	247	2.94	0.004 ***	0.37
	Less than once a day	130	4.1	0.8				
It helps me maintain my health.	Once a day or more	119	4.2	0.8	247	2.58	0.01 *	0.33
	Less than once a day	130	3.9	0.9				
It is helpful for the health of dog(s).	Once a day or more	119	4.5	0.6	247	2.58	0.01 *	0.33
	Less than once a day	130	4.3	0.8				
If I don’t take a dog(s) walk often, I feel guilty.	Once a day or more	119	4.4	0.8	247	2.51	0.013 *	0.32
	Less than once a day	130	4.2	0.8				
During the pandemic period, I walked my dog(s) more often.	Once a day or more	119	3.5	1.2	237	2.80	0.006 **	0.36
	Less than once a day	130	3.1	1.1				

* *p* < 0.05, ** *p* < 0.01, *** *p* < 0.001.

**Table 5:** Correlation between dog walking, loneliness, and attachment.

	Dog Walking	Loneliness	Attachment
Dog walking	1		
Loneliness	0.105	1	
Attachment	0.481 **	0.226 **	1

** *p* < 0.01, two-tailed.

**Table 6:** Bootstrapping results on the mediator effect.

Effect	B	SE	*t*	*p*	LL 95% CI	UL 95% CI
a	0.454	0.053	8.616	0.000	0.340	0.557
b	0.124	0.037	3.635	0.000	-	-
a × b	0.125	0.039	3.219	0.002	0.049	0.202
c	0.054	0.033	1.644	0.102	−0.011	0.118
Total effect	0.054	0.033	−0.082	0.102	−0.011	0.118
Direct effect (c)	−0.003	0.037	1.644	0.934	−0.075	0.069
Indirect effect (a × b)	0.057	0.018	-	-	0.023	0.096

## Data Availability

The data presented in this study are available on request from the authors.

## Supplementary Materials

The following are available online at https://www.mdpi.com/article/10.3390/ani12040483/s1, File S1: The questionnaire: Questionnaire survey on dog attachment and dog walking.
