# Supplementary material for: Influences of Dog Attachment and Dog Walking on Reducing Loneliness during the COVID-19 Pandemic in Korea
Source: Animals (Basel). 2022 Feb 16;12(4):483. doi: 10.3390/ani12040483 (PMC8868061; doi:10.3390/ani12040483)
Supplement: Supplementary file 1 [file animals-12-00483-s001.zip › animals-1558476-supplementary File S1.pdf]

Dog Attachment and Dog Walking During the COVID-19 Pandemic

The purpose of our study is to investigate the changes in dog attachment and dog walking during the COVID-19 pandemic and the effects of dog attachment and dog walking on dog owners' loneliness. The results of this survey will be used only for research purposes. Please feel free to contact us (thdwlsrud001@naver.com) if you have any questions. Thank you.

- Demographic Characteristics

1. What is your gender?

   ① Female ② Male

2. What is your age?

   ① 10-20s ② 30s ③ 40s ④ 50s ⑤ 60s ⑥ 70-80s

3. What is your family type?

   ① Single residence ② Married couple ③ Living with parents ④ Living with children

4. How much do you earn?
5. What is your level of education?

   ① Under the high school graduate ② College graduate ③ Postgraduate school graduate

6. What is your job type?
7. What type of home do you live in?

   ① Apartment ② Rowhouse/ Multi house ③ Detached house ④ Etc

8. What do you think about your health?

   ① Very good ② Good ③ Average ④ Poor ⑤ Very poor

9. Where do you live?

   ______-si ______-gu ______-dong

A. About your companion dog

A1. How big is your dog?

	Small dog (10kg or less)	Medium dog (10–25kg)	Large dog (25kg and over)
Dog 1			
Dog 2			
Dog 3			

A2. How long have you breed your dog?

About ___ year

A3. Please answer the following questions about your relationship with dog(s).

		Strongly disagree				Strongly agree
1.	I think of my dog(s) as a family.	1	2	3	4	5

2.	I feel happier thanks to my dog(s).	1	2	3	4	5
3.	I feel less lonely thanks to my dog(s).	1	2	3	4	5
4.	I talk to my dog(s).	1	2	3	4	5
5.	My dog(s) seems to know my feelings well.	1	2	3	4	5
6.	I often play with my dog(s).	1	2	3	4	5
7.	During the pandemic, I spend more time with my dog(s).	1	2	3	4	5
8	During the pandemic, I became more attached to my dog(s).	1	2	3	4	5

B. About dog(s) walking

B1. How many times did your dog go out for a walk?

______ a week, or _____ a month, or ___ none (reason: _________)

B2. Who do you walk with your dog in the household?

① Myself ② Parents ③ A marriage partner ④ Children ⑤ Etc

B3. How long do you take a walk with your dog(s)?

① 30 minutes or less ② More than 30 minutes–1 hour ③ More than 1–2 hours ⑤ More than 2 hours

B4. Where do you take a walk with your dog(s)?

	Place name	Reason
Neighborhood		
Park		
Riverside trail		
Etc		

B5. Please answer the following questions about your perception of dog walking

		Strongly disagree				Strongly agree
1.	I also get to do more physical activities.	1	2	3	4	5
2.	It helps me maintain my health.	1	2	3	4	5
3.	It is helpful for the health of dog(s).	1	2	3	4	5
4.	It's a break for me.	1	2	3	4	5
5.	Opportunities to talk to other people arise.	1	2	3	4	5
6.	If I don't take a dog(s) walk often I feel guilty.	1	2	3	4	5
7.	During the pandemic, I walked my dog(s) more often.	1	2	3	4	5
8	During the pandemic, I felt that there was not enough space to take a walk with my dog(s).	1	2	3	4	5
9.	During the pandemic, I felt the importance of the park/green area to take a walk.	1	2	3	4	5

Thank you for your cooperation.
